# Eco-evolutionary dynamics of experimental *Pseudomonas aeruginosa* populations under oxidative stress

**DOI:** 10.1099/mic.0.001396

**Published:** 2023-11-09

**Authors:** Taoran Fu, Danna R. Gifford, Christopher G. Knight, Michael A. Brockhurst

**Affiliations:** ^1^​ Division of Evolution, Infection and Genomic Sciences, School of Biological Sciences, Faculty of Biology, Medicine and Health, The University of Manchester, Manchester M13 9PT, UK; ^2^​ Department of Earth and Environmental Sciences, School of Natural Sciences, Faculty of Science and Engineering, The University of Manchester, Manchester M13 9PT, UK

**Keywords:** eco-evolutionary dynamics, oxidative stress, reactive oxygen species, *Pseudomonas aeruginosa*, cystic fibrosis, bronchiectasis

## Abstract

Within-host environments are likely to present a challenging and stressful environment for opportunistic pathogenic bacteria colonizing from the external environment. How populations of pathogenic bacteria respond to such environmental challenges and how this varies between strains is not well understood. Oxidative stress is one of the defences adopted by the human immune system to confront invading bacteria. In this study, we show that strains of the opportunistic pathogenic bacterium *Pseudomonas aeruginosa* vary in their eco-evolutionary responses to hydrogen peroxide stress. By quantifying their 24 h growth kinetics across hydrogen peroxide gradients we show that a transmissible epidemic strain isolated from a chronic airway infection of a cystic fibrosis patient, LESB58, is much more susceptible to hydrogen peroxide than either of the reference strains, PA14 or PAO1, with PAO1 showing the lowest susceptibility. Using a 12 day serial passaging experiment combined with a mathematical model, we then show that short-term susceptibility controls the longer-term survival of populations exposed to subinhibitory levels of hydrogen peroxide, but that phenotypic evolutionary responses can delay population extinction. Our model further suggests that hydrogen peroxide driven extinctions are more likely with higher rates of population turnover. Together, these findings suggest that hydrogen peroxide is likely to be an effective defence in host niches where there is high population turnover, which may explain the counter-intuitively high susceptibility of a strain isolated from chronic lung infection, where such ecological dynamics may be slower.

## Introduction

For opportunistic pathogenic bacteria colonizing from environmental reservoirs, the human host likely represents a hostile and stressful novel environment, albeit one rich in resources. In particular, many infection sites contain elevated amounts of reactive oxygen species (ROS) generated by the host inflammatory response, including both acute [1] and chronic infections [2]. A variety of host cells, including macrophages and neutrophils, produce ROS at infection sites [3]. Recent studies also show that ROS generated by neutrophils is further enhanced in the lungs of COVID-19 patients [4], potentially causing post-COVID bacterial secondary infections to experience even greater ROS stress [5]. Hydrogen peroxide is one of the key ROS produced by host cells, and it is also commonly present in the natural environment [6–8]. Many microbes generate hydrogen peroxide during competitive interactions [9, 10], and it is frequently used in disinfectants for cleaning surfaces [11]. Hydrogen peroxide causes damage to bacteria by reacting with iron centres in microbial enzymes and by forming toxic hydroxyl radicals through the Fenton reaction [12]. Additionally, the Fenton reaction is affected by environmental pH [13], which varies *in vivo* [14].

*Pseudomonas aeruginosa* is widespread in the natural environment [15] and causes a range of opportunistic infections, including the airways of people with cystic fibrosis (CF) [16, 17] and bronchiectasis [18]. *P. aeruginosa* has a large, flexible genome [19] encoding multiple global regulatory and quorum-sensing (QS) systems that enable it to respond to changing physiological conditions [20, 21], including exposure to ROS [22]. *P. aeruginosa* encodes several catalases and peroxidases to protect cells from oxidative stress. KatA is a constitutively expressed extracellular catalase, whereas KatB production is induced by hydrogen peroxide and KatC production by temperature [23]. Peroxidases such as glutathione peroxidase (Gpx), thiol peroxidase homolog (Tpx) and alkyl hydroperoxide reductase (AhpC) are also believed to enhance *P. aeruginosa* tolerance against ROS, including H_2_O_2_ [23, 24]. OxyR is the central regulator for hydrogen peroxide detoxifying systems, including controlling catalase expression, with additional layers of regulation by quorum-sensing systems, *lasRI*/*rhlRI* [25, 26], and the stringent response regulator DksA [27]. Importantly, different strains of *P. aeruginosa* exhibit different basal levels of expression of the main catalase gene, *katA*, resulting in differences in susceptibility to hydrogen peroxide. For instance, among the commonly used lab strains, PA14 exhibits lower *katA* expression and higher hydrogen peroxide susceptibility than PAO1 [23, 28].

The impact of variation in hydrogen peroxide susceptibility between diverse *P. aeruginosa* strains on population dynamics under sustained oxidative stress in not well understood. Here, we quantified the effect of hydrogen peroxide on the growth kinetics of three *P. aeruginosa* strains, specifically the lab strains PA14 and PAO1, and a clinical strain previously isolated from a CF chronic lung infection, LESB58. We next tracked the longer-term population dynamics of PAO1 and PA14 with or without sustained subinhibitory hydrogen peroxide at two pH levels representative of the range of pH-levels in lung sputum [14, 29], and create a mathematical model parameterized for this system. We show, unexpectedly, that LESB58 is highly susceptible to hydrogen peroxide despite having been isolated from a chronic CF lung infection, an environment where ROS-levels are likely to be elevated. We further show that sustained oxidative stress drives population extinction of PA14 but not PAO1, consistent with PA14’s higher susceptibility to hydrogen peroxide. We show that PA14 serially passaged in elevated levels of H_2_O_2_ did increase in their ability to grow under oxidative stress conditions, but not to the extent of wild-type PAO1, and sufficiently only to delay but not prevent extinction. Using the mathematical model, we further show that oxidative stress driven extinctions are more likely in systems with higher population turnover, potentially explaining why highly susceptible strains, such as LESB58, persist in chronic infections despite the high levels of ROS.

## Methods

### Bacteria and culture condition

We used the *P. aeruginosa* strains PAO1, PA14 and LESB58. PAO1 was originally isolated from a hospital in Australia in 1955 [30], with subsequent adaptation under laboratory conditions [31, 32]. PA14 was isolated from a burn wound in a hospital in 1977 [33] but its high virulence to plants suggests it may have originated from a plant or soil environment [34]. Liverpool Epidemic Strain LESB58 was isolated from a CF lung infection [35]. Bacteria were grown in a defined medium mimicking CF sputum, Synthetic Cystic Fibrosis Sputum Medium (SCFM, pH=6.8) [36] and various modified versions of this medium designed to mimic environmental stresses experienced during infections. For the H_2_O_2_ susceptibility experiments, we supplemented the SCFM with various concentrations of H_2_O_2_ to create an oxidative stress gradient (concentrations of H_2_O_2_ were approximately as follows: 0, 0.03, 0.06, 0.12, 0.24, 0.5, 1, 2, 4, 8, 16, 31, 63, 125 mM). For the serial passage experiment, we supplemented SCFM with either H_2_O_2_ to reach a final concentration of 2 mM (SCFM-Ox), 1 M HCl to adjust pH to 5.4 (SCFM-Ac), or both H_2_O_2_ and 1 M HCl to achieve a final concentration of 2 mM H_2_O_2_ and pH to 5.4 (SCFM-Ox-Ac). Certain ingredients, H_2_O_2_, HCl and a final concentration of 3.6 µM Fe(II), were added freshly and filter sterilized on the day of use. To compare growth in SCFM with lab broth, we also tested the growth kinetics in King’s B medium (KB) [37]. Overnight cultures were incubated in 200 µl of medium in wells of 96-well plates at 37 °C in a humidity-controlled incubator, with 80 % humidity.

### Growth kinetics to assess H_2_O_2_ susceptibility

All growth kinetics were set up from bacterial overnight cultures (inoculum optical density OD=0.7~0.9) by transferring 1 % of these cultures into wells of 96-well plates containing 200 µl of medium. Then, 96-well plates were sealed using gas-permeable membranes (Breathe-Easy sealing membrane, Sigma-Aldrich, USA) to avoid cross-contamination during shaking. Growth kinetics were measured using a microplate reader (LogPhase 600, Biotek, USA) measuring absorbance at 600 nm every 20 min. Bacteria were grown under shaking (orbital, 800 r.p.m.) at 37 °C for 24 h. We performed six replicate growth curves per strain (PAO1, PA14, LESB58) across a gradient of oxidative stress levels.

### Serial passage experiment

Nine independent colonies each of PAO1 and PA14 were picked and resuspended in phosphate-buffered saline (PBS) and inoculated into fresh SCFM media and grown overnight. About 10^7^ cells per overnight culture were used to found one of nine independent replicate populations in each of four selection treatments, which were SCFM, SCFM-Ox, SCFM-Ac, SCFM-Ox-Ac. Replicate populations were propagated by 1 % daily serial transfer for 12 days (~80 generations) in 96-well plates wherein each well contained 200 ul of the relevant medium. Cultures were grown for at least 22 h between transfers. Before each transfer, a point reading of absorbance at 600 nm was measured using a microplate reader (LogPhase 600, Biotek, USA). Population samples were stored daily for 7 days and every 2 days thereafter as glycerol stocks at −80 °C.

### Growth kinetics of evolved PA14

Because we observed population extinctions of PA14 in the SCFM-Ox serial transfer experiment, we tested the growth responses of each replicate PA14 population from the SCFM and SCFM-Ox treatments sampled on days 4, 6 and 8. We obtained 24 h growth kinetics for six random clones per population grown either in SCFM or SCFM-Ox growth media using a microplate reader (LogPhase 600, Biotek, USA) measuring absorbance at 600 nm every 20 min. Bacteria were grown under shaking (orbital, 800 r.p.m.) at 37 °C for 24 h.

### Statistical analysis

Growth kinetics were analysed using R (4.2.0) where the maximum growth rate (Absorbance × h^−1^), maximum absorbance as endpoint population density, integral (area under the growth curve), and lag time were determined using the same method as a previous study [38]. Lag times longer than 24 h were recorded as 24 h rather than an infinite value for the statistical analysis if not specified. In addition, the growth rate was also calculated using R function ‘all_splines’ from the package ‘growthrates’ as the maximum intrinsic growth rate (mumax) from the smoothed curve. Statistical analysis including t-test, analysis of variance (ANOVA), analysis of covariance (ANCOVA), survival analysis, linear mixed-effect model, principal component analysis (PCA) and data visualization was performed in R (4.2.0). Linear mixed-effect model of phenotypic adaptation of PA14 to SCFM or SCFM.Ox was run with lineage as random effect. Evolution trajectory analysis followed a previous statistical model of multivariate phenotypic change trajectory analysis [39].

### Mathematical model

To better understand our experimental results, we modified an existing model of bacterial growth to include variable susceptibility to oxidative stress. We simulated bacterial growth using the Baranyi and Robert model (1994) [40] in Matlab R2022a, adding a new term to describe susceptibility to H_2_O_2_:

(1) $$\frac{dN}{dt}=g(N,t,K,r_{max},l)\times (1-\beta \times c_{H_{2}O_{2}})$$

(2) $$\frac{dc_{H_{2}O_{2}}}{dt}=-\frac{K_{cat}\times \rho \times c_{H_{2}O_{2}}}{K_{m}+c_{H_{2}O_{2}}}\times N-b\times c_{H_{2}O_{2}}$$

Equation 1 is the growth rate of bacteria and equation 2 is the decomposition rate of H_2_O_2_ in the bacterial culture. Among these equations, *N* represents the abundance of bacteria, *t* is time, *K* is the maximum capacity, *r*_max_ is the maximum growth rate, is the lag time, where *g*(*N*,*t*,*K*,*r*_max_,*l*) is the Baranyi and Robert logistic growth model with lag phase [40], *β* represents the bacterial growth inhibition triggered by H_2_O_2_, *c*_H2O2_ represents the concentration of H_2_O_2_. In equation 2, *K*_m_ is the Michaelis constant and *K*_cat_ is the maximum rate of catalase degradation for the amount of catalase generated by a unit of bacteria, *ρ*, and multiplied by the amount of bacteria present in the culture, *N; b* is the self-decomposition rate of H_2_O_2_ at incubation temperature 37 °C. To explore the impact of H_2_O_2_ on bacterial ecological dynamics, we took the bacterial growth inhibition triggered by H_2_O_2_, *β*, and the amount of catalase generated by a unit of bacteria, *ρ*, from the growth of PA14 as a baseline (Table S1, Figs S1 and S2). We then varied them by simultaneously multiplying *β* and dividing *ρ* by the same value, which is reported as a fold change in sensitivity to H_2_O_2_ relative to PA14. Using this differential equation model, we also simulated a 1 % daily serial transfer experiment varying the concentration of H_2_O_2_ and the sensitivity of bacteria to H_2_O_2_ stress, enabling us to generalize our findings across a wider range of values than was possible experimentally. The extinction rate is defined as the reciprocal of the day on which extinction occurred, with a maximum value, 1, suggesting an immediate extinction on the day of inoculation and a minimum value, 0, as survival after the 12 day transfer. The mathematical model is parameterized based on experimental bacterial growth in this study and other references (see Table S1, available in the online version of this article). To further investigate the mismatch in extinction rate between our first simulation and serial passage experiment, we modified the sensitivity of the strain to H_2_O_2_ beginning on day 6 to account for evolution using parameter values matching the measured bacterial growth kinetics of evolved PA14 (see Fig. S3). We then performed additional simulations wherein we systematically varied the serial transfer dilution rate to mimic different levels of population turnover to explore how this affected extinction rate.

## Results

### Diverse *P. aeruginosa* strains vary in their susceptibility and growth responses to H_2_O_2_

**Fig. 1.**
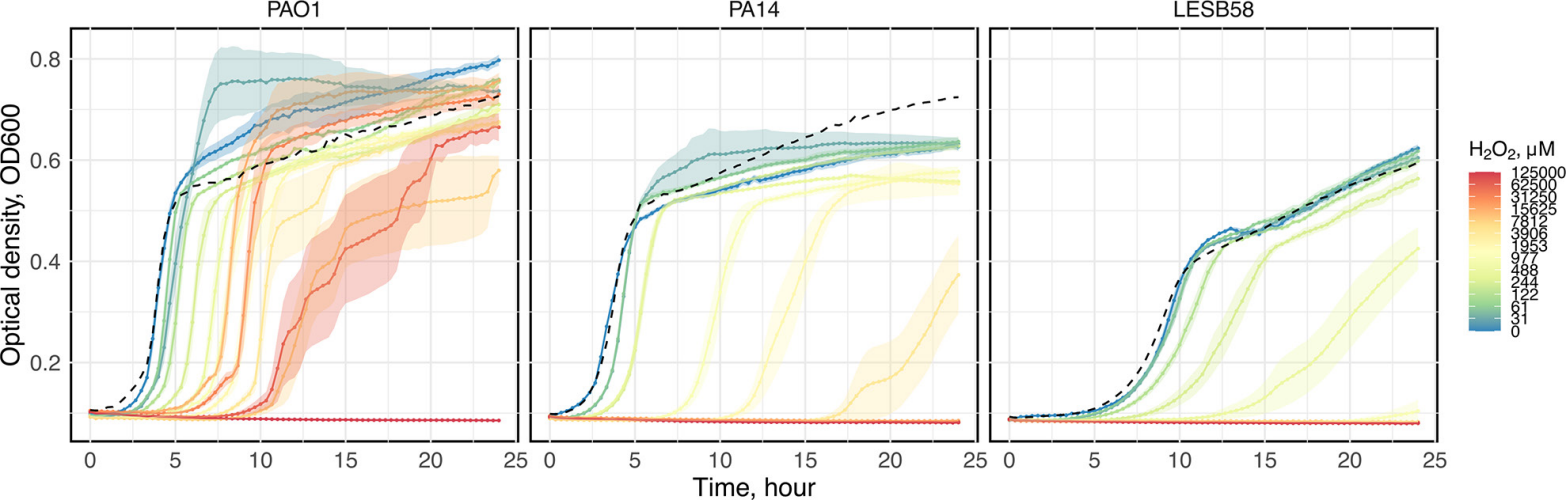
Hydrogen peroxide (H_2_O_2_) has differential effects on growth of *P. aeruginosa* isolates PAO1, PA14 and LESB58. Higher concentrations of H_2_O_2_ have a greater inhibitory effect on growth. Solid lines indicate the average of optical density at 600 nm (OD600) and ribbons indicate the standard error across six replicates; dashed lines indicate growth kinetic in KB medium of each strain. Colours denote the concentration of H_2_O_2_ in SCFM as explained in the visual key.

The response of growth in different H_2_O_2_ concentration varied across strains (Fig. 1; mixed-effect model, three-way interaction between time, H_2_O_2_ concentration and strain with absorbance as response variable, *χ* ^2^_2_=10.928, *P*=0.0042). Lag phase, but not maximum growth rate or maximum population density, was significantly affected by H_2_O_2_ concentration in a strain-specific manner (nongrowing populations excluded, two-way ANOVA, H_2_O_2_ concentration by strain interaction, lag phase: *F*_2,213_=3.756, *P*=0.0249; maximum growth: *F*_2,213_=2.879, *P*=0.0583; maximum optical density: *F*_2,213_=1.290, *P*=0.2774). Specifically, increasing concentration of H_2_O_2_ progressively extended lag phase, eventually completely supressing growth, and this effect was strongest in LESB58 and weakest in PAO1. Accordingly, the degree of growth inhibition by H_2_O_2_ at 24 h varied among strains (two-way ANOVA, endpoint OD, *F*_2,242_=95.623, *P*<0.0001), with LESB58 being the most susceptible, followed by PA14, while PAO1 was the least susceptible. Together these data show that *P. aeruginosa* strains differ significantly in their susceptibility and growth response to oxidative stress.

### Contrasting ecological dynamics of PAO1 and PA14 under sustained oxidative stress

**Fig. 2.**
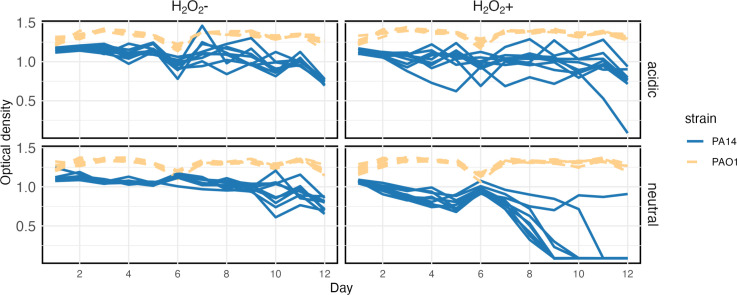
Contrasting population dynamics between PA14 and PAO1 strains. Population density over time in a 12 day serial passage determined by optical density.

**Fig. 3.**
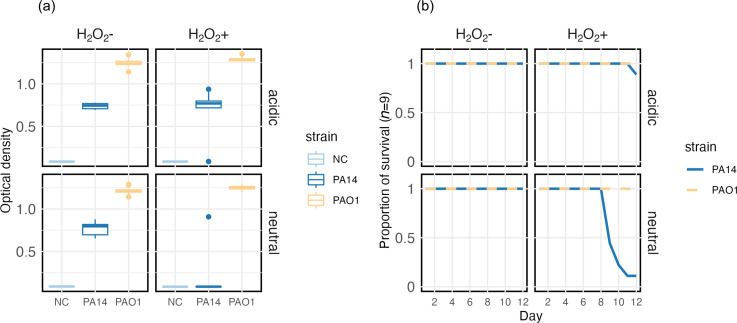
Contrasting population dynamics between PA14 and PAO1 strains. (a) Optical density at the end of the 12 day passage. NC indicates the negative controls. (b) Proportion of surviving replicates per treatment over time. Plot panels are faceted by treatment as denoted by labels. Colours denote bacterial strains PAO1 (orange) and PA14 (blue) or negative controls (light blue).

To investigate whether differences in short-term susceptibility to H_2_O_2_ predict longer-term population survival under sustained oxidative stress, we performed a serial passage experiment wherein PAO1 and PA14 were propagated with or without subinhibitory H_2_O_2_ at two pH-levels (6.8 and 5.4) representative of the range that can be observed in lung sputum [14, 29]. Whereas PAO1 populations survived under all conditions, population extinctions were observed for PA14 under oxidative stress (Figs 2 and 3a, b, *χ* ^2^_1_=35.6, *P*<0.0001) with more extinctions occurring in neutral compared to acidic pH conditions (*χ* ^2^_1_=33, *P*<0.0001). Accordingly, end-point population densities differed significantly across strains and treatments (Figs 2 and 3a) (two-way ANOVA, *F*_10,102_=36.575, *P*<0.0001). Together these data show that higher susceptibility to oxidative stress reduced longer-term population survival at subinhibitory levels of H_2_O_2_ and that this effect was exacerbated at higher pH for PA14.

### Modelling shows that short-term susceptibility predicts extinction dynamics

**Fig. 4.**
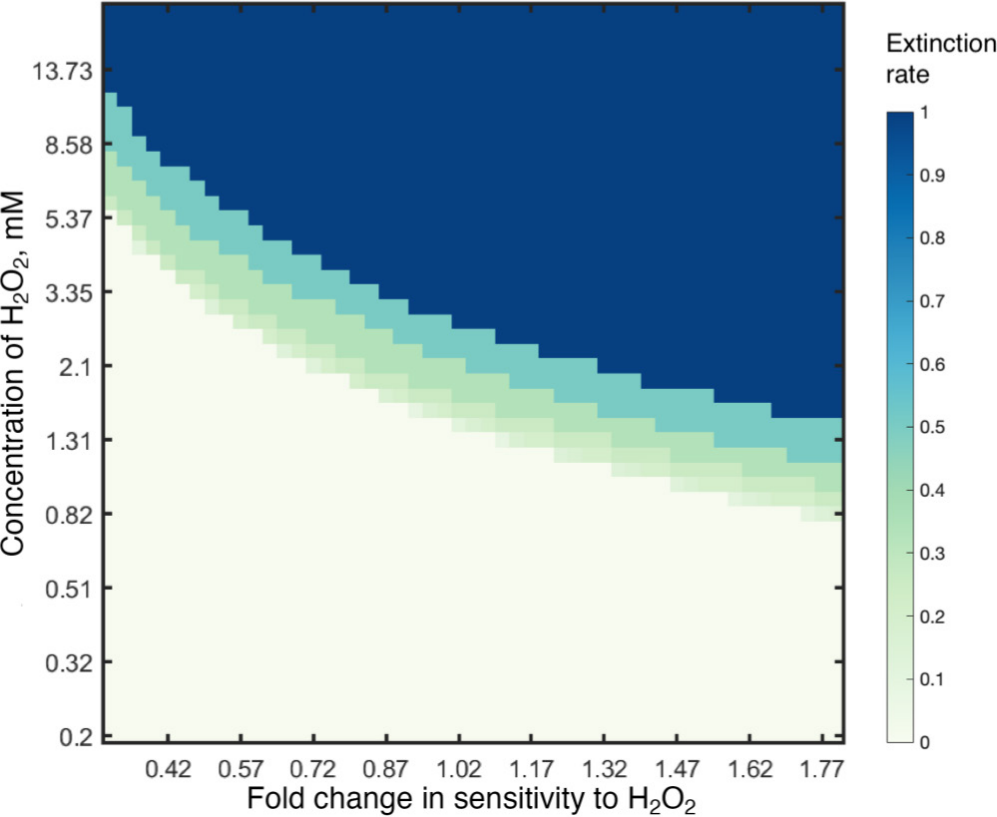
Extinction rate is shown in colours plotted as a function of fold change of sensitivity to H_2_O_2_ relative to wild-type PA14 and the concentration of H_2_O_2_ in a mathematical model with a 1 : 100 serial daily transfer.

To better understand the contrasting ecological dynamics of PAO1 and PA14 under oxidative stress and to generalize our findings across a wider range of parameters we modified an existing mathematical model of bacterial growth [40]. We modelled bacterial populations growing under a serial passage regime allowing growth to be a function of the concentration of hydrogen peroxide to model variable susceptibility, as we observed in the growth kinetics (parameters and simulations of growth compared to experimental data are shown in the Supplementary Material 1). Consistent with our serial transfer experimental results, the model predicts that the probability of population extinction increases as a function of bacterial sensitivity to H_2_O_2_ and the environmental concentration of H_2_O_2_ (Fig. 4). Parameterizing the model for ancestral PA14 (Fig. S1), shows that subinhibitory levels of oxidative stress are sufficient to cause population extinction because the population growth is insufficient to overcome the 1 : 100 daily serial dilution (Fig. S2). Interestingly, however, our model predicts faster PA14 extinction than observed in the serial passage experiment, with extinctions 3 days earlier (Figs 2 and S2b). This mismatch suggests that the PA14 populations may have adapted to the oxidative stress conditions to prolong persistence, albeit not sufficiently to prevent eventual extinction.

### Adaptation of PA14 to subinhibitory H_2_O_2_

**Fig. 5.**
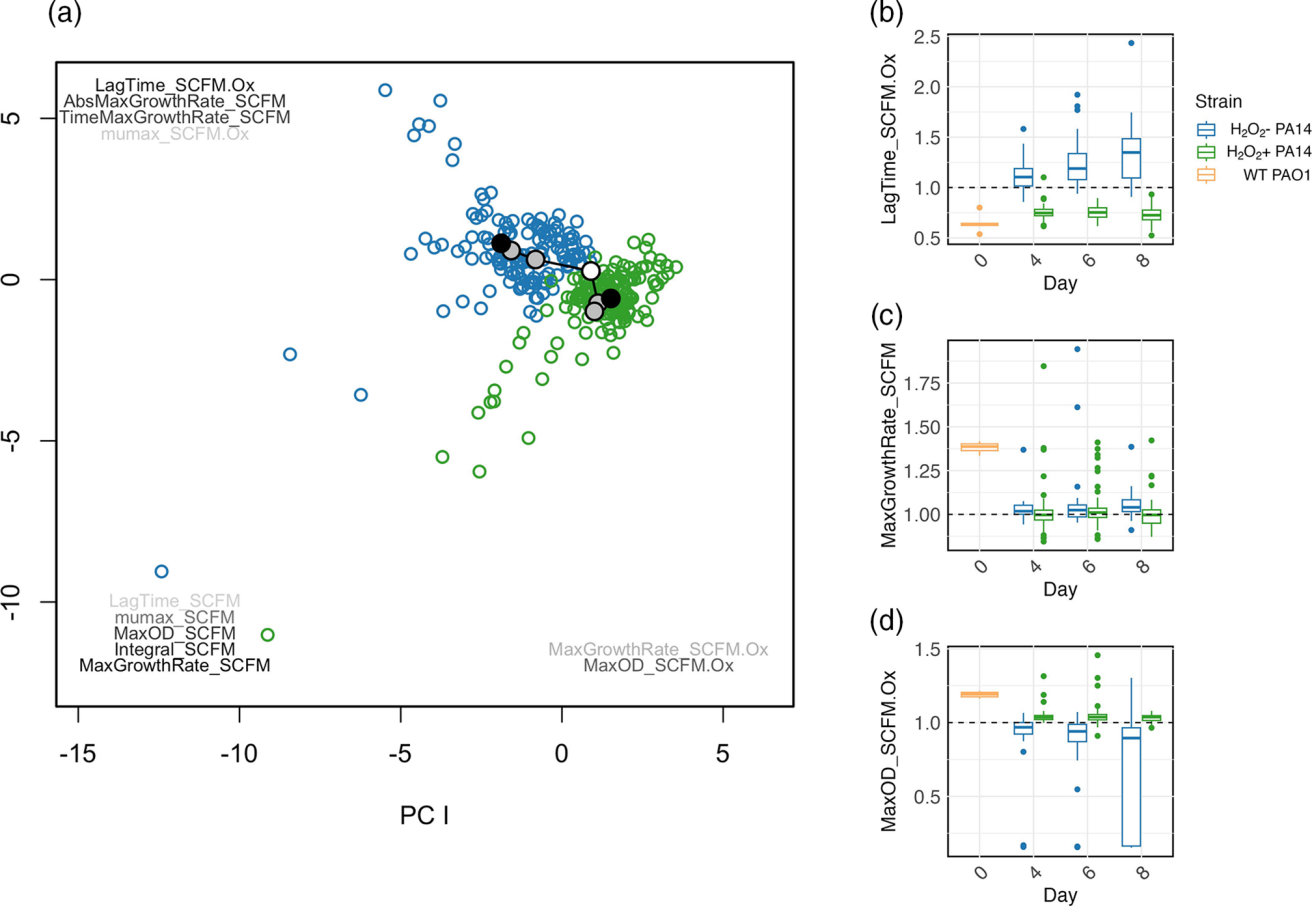
Phenotypic adaptation of evolved PA14 varies across treatments. (a) PCA analysis of phenotypic growth parameters of evolved PA14 clones relative to wild-type PA14. Closed circles represent the mean of six clones per population on day 0 (white), 4, 6 (grey) and 8 (black); open circles show individual clones evolved in SCFM (blue) or SCFM_Ox (green). Variables are coloured from black to light grey indicating their weighting and positioned by their direction with a suffix indicating whether determined experimentally without (SCFM) or with H_2_O_2_ (SCFM_Ox). (b, c and d) The relative growth parameters of highest weight in each direction from the PCA analysis of either evolved PA14 clones from SCFM (blue) or SCFM_Ox (green) treatments or wild-type PAO1 (orange), with dashed line at value 1.0 indicating equality with ancestral PA14.

The mismatch between our modelling and experimental results suggests that PA14 populations in SCFM-Ox may have adapted to the oxidative stress conditions. To test this, we measured the 24 h growth kinetics in SCFM and SCFM-Ox media of PA14 colonies isolated on days 4, 6 and 8 of the serial transfer experiment from replicate populations within the SCFM and SCFM-Ox treatments. The phenotypic evolutionary trajectories of populations selected under these contrasting treatments differed both in terms of direction and rate (Fig. 5a; trajectory analysis, *p*_treatment_ <0.001, *p*_time_=0.002, and *p*_interaction_ <0.001). Indeed, whereas PA14 selected in SCFM-Ox acquired a shortened lag time in the SCFM-Ox test media, the opposite pattern occurred in PA14 selected in SCFM (Fig. 5b, d; mixed-effect model, interaction by day by treatment, for relative lag time *χ* ^2^_1_=14.096, *P*=0.0002, for relative maximum OD *χ* ^2^_1_=19.856, *P*<0.0001). In contrast, PA14 selected in SCFM acquired an increased maximum growth rate in SCFM, which did not occur in PA14 selected in SCFM-Ox (Fig. 5c; mixed-effect model, *χ* ^2^_1_=4.2201, *P*=0.0399). These data suggest that PA14 adapted to oxidative stress in the SCFM-Ox treatment by reducing lag time, but, conversely, that adapting to SCFM per se increased susceptibility to oxidative stress, suggesting an evolutionary trade-off.

### Modifying the model to reflect evolutionary changes in susceptibility

**Fig. 6.**
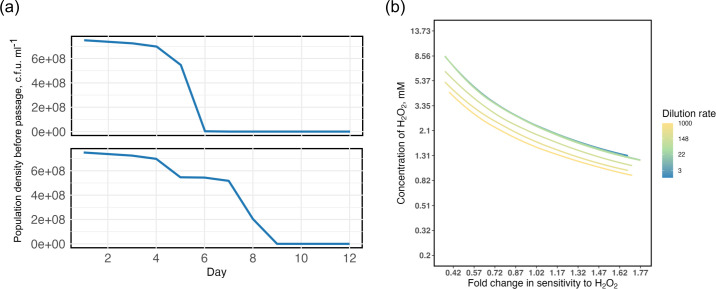
Population dynamics and boundaries of extinction events in the mathematical model. (a) Simulation of population density in a 1 : 100 serial passage parameterized for ancestral PA14 (top panel; fold change in sensitivity to H_2_O_2_ relative to PA14=1) or evolved PA14 (bottom panel; fold change in sensitivity to H_2_O_2_=0.96; from day 6). (b) Boundaries of extinction events at different serial dilution rates shown in colours plotted as a function of fold change in sensitivity to H_2_O_2_ relative to wild-type PA14 and the concentration of H_2_O_2_.

To test if the experimentally observed adaptation of PA14 to oxidative stress could explain the mismatch between the extinction dynamics in our model versus experimental results, we re-ran the model with two stages, where the later stage that began on day 6 had parameters matching the growth kinetics of adapted strains (Fig. S3). The new growth kinetic parameters prolonged population survival in the model, and substantially improved the fit of the temporal dynamics between the simulation and the experiment (Fig. 2 lower-right and Fig. 6a). This suggests that the acquired reduced susceptibility to ROS was sufficient to delay, but not to prevent extinction.

### Generalizing the model for a wider range of population turnover rates

It is likely that population turnover rates in chronic CF lung infections are not as high as those in our serial transfer experiment, which we predicted may help to explain why the clinical strain we tested, LESB58, has not evolved reduced susceptibility to oxidative stress despite prolonged exposure to ROS from host immunity. Using our mathematical model, we varied the dilution rate per serial transfer to mimic systems with different population turnover rates. Indeed, lower serial dilution rates, and thus lower rates of population turnover, do enable more highly susceptible bacterial populations to survive at higher environmental concentrations of H_2_O_2_ (Fig. 6b).

## Discussion

Upon colonizing a human host, opportunistic bacterial pathogens are likely to experience a challenging and stressful environment, but our understanding of how these stressors impact eco-evolutionary responses of bacterial populations is limited. Here we show that strains of *P. aeruginosa* vary in their susceptibility to oxidative stress. Increasing concentrations of hydrogen peroxide progressively extended lag-phase, eventually completely suppressing bacterial growth. It is probable that this effect reflects the differential expression of oxidative stress defence mechanisms among these strains, affecting their ability to detoxify their environment, which is consistent with known differences in *katA* expression between PAO1 and PA14 [23, 28]. The much higher sensitivity of LESB58 is less well explained from a mechanistic perspective. One possible explanation is that hydrogen peroxide may trigger prophage induction in LESB58 [41]. Hydrogen peroxide is a well-known inducer of the phage lytic cycle [42–45] and the LESB58 genome contains five active prophages that are known to retain lytic activity during chronic CF lung infection [46]. More detailed mechanistic analysis will be required to determine the molecular mechanism causing the variable sensitivity to hydrogen peroxide across strains. We further show, using a combination of serial transfer experiments and mathematical modelling, that short-term susceptibility to oxidative stress predicted long-term survival of populations against sustained exposure to subinhibitory oxidative stress. However, rapid phenotypic evolution of reduced susceptibility to oxidative stress in PA14 altered these population dynamics, prolonging survival of populations but ultimately was insufficient to prevent extinction, which is consistent with previous studies in *E. coli* [47, 48]. This heritable change in susceptibility to oxidative stress could be due to mutation [26, 49] or phenotypic plasticity [50, 51]. Finally, using our mathematical model we predict that the rate of population turnover is critically important in determining whether oxidative stress causes population extinction, with lower population turnover promoting survival of more highly susceptible strains.

By quantifying how oxidative stress impacts bacterial growth kinetics, we were able to understand how oxidative stress affects the longer-term ecological dynamics of bacterial populations. We show a critical interaction between ROS-mediated growth inhibition, through extending lag phase, and the rate of population turnover, that together control extinction probability. As a consequence, even subinhibitory levels of ROS can drive extinction of bacterial populations in environments or niches with higher population turnover. The actual level of ROS experienced by bacteria in the respiratory tract is unclear due to challenges of accurately quantifying this *in situ*, however it is likely to vary between body sites and even spatiotemporally within organs [52–55]. We predict, therefore, that ROS defences may be more effective at lower concentrations in host niches with higher turnover, such as the bladder or gut [56, 57]. In contrast, in chronic lung infection it is likely that *P. aeruginosa* experiences relatively lower rates of population turnover [58, 59], which may result in reduced selection to maintain potentially costly low-level sensitivity to oxidative stress [60, 61]. Although we did not assay enough strains to test this hypothesis, our data are consistent with this idea: LESB58 (isolated from a chronic infection [35]), is far more susceptible to oxidative stress than either PAO1 or PA14 (isolated from acute infections [30, 33]). Note that this is not sufficient evidence to show that *P. aeruginosa* phylogroups vary systematically in their oxidative stress sensitivity. More work will be needed to quantify susceptibility to oxidative stress across diverse *P. aeruginosa* strains isolated from a wider range of niches.

Our findings add to a growing body of studies combining ecological modelling and experiments that show the importance of accounting for phenotypic evolutionary dynamics to fully understand the dynamics of bacterial populations [62–66]. Here, acquisition of reduced susceptibility to oxidative stress by PA14 prolonged population survival. Our analysis of adapted phenotypes further revealed a potential evolutionary trade-off between adapting to oxidative stress and adapting to the sputum-mimicking medium. Such trade-offs are consistent with previous findings that adaptation to stressors can have costly pleiotropic effects on bacterial growth in stressor-free environments. For instance, oxidative stress selects for mutants upregulating the *wsp* system, resulting in rough small colony variants (RSCVs) that grow slower in the absence of exogenous ROS [49]. Acquired resistance to antibiotics is also usually costly in the absence of drug [67, 68]. Adaptation to other environmental stressors including soil nickel, detergent and osmotic stress suggests a trade-off between adapting to the stressor and normal growth rate in a stressor-free environment [69]. Fitness trade-offs between contrasting environments are likely to drive the evolution of specialists, here leading to variants that are good at exploiting the sputum environment or at resisting ROS, but which cannot do both. This may limit the success of low-sensitivity variants, if levels of oxidative stress are variable in space or time at host infection sites.

Oxidative stress is an important component of host defence against infecting pathogens, but, as shown here, susceptibility to oxidative stress varies greatly between *P. aeruginosa* strains. Our data suggest that such variation in susceptibility to oxidative stress may be explained by differences in population ecology among host niches and/or by evolutionary trade-offs whereby adaptation to oxidative stress constrains adaptation to the other components of the within-host environment or vice versa.

## Supplementary Data

Supplementary material 1
