# Supplementary material for: Eco-evolutionary dynamics of experimental Pseudomonas aeruginosa populations under oxidative stress
Source: Microbiology (Reading). 2023 Nov 9;169(11):001396. doi: 10.1099/mic.0.001396 (PMC10710836; doi:10.1099/mic.0.001396)
Supplement: Supplementary material 1 [file mic-169-1396-s001.pdf]

Supplementary figures and tables

Table S1. Parameterisation of bacterial growth model challenged by OS.

**Growth equation (Equation 1)**	**Parameter(s)**	**Notes**	
	r_{max}, l, K	Baranyi model fit of PA14 and PAO1 growth kinetics using \`nlsmicrobio\` R package.	
	β_{PA14}	Simulated based on PA14 growth kinetics with a Pearson correlation coefficient (see Fig S1). Other strains β were simulated by simultaneously multiplying β_{PA14} and dividing ρ_{PA14} by the same value.	
	N_0, K(or N_{max})	Estimation from planktonic growth of PA14 and PAO1	
**Michaelis-Menten equation (Equation 2)**	**Parameter(s)**	**value**	**Reference(s)**
	K_{cat}	2.5576×10^8 h^{-1}	(1)
	K_m	67 mM	(1)
	ρ_{PA14}	5×10^{-18} mol $cell^{-1}$	(1–3)
**H_2O_2 natural decomposition (Equation 2)**	**Parameter(s)**	**value**	**Reference(s)**
	b	0.0289 h^{-1}	(4)

Fig S1. Simulation of bacterial growth of PA14 ($\beta_{PA14} = 889$) (A) and PAO1 (fold change in sensitivity to H_2O_2 relative to PA14 = 0.90) (B) in 2 mM H_2O_2 environment. Both simulation data at the chosen parameters (light purple) and experimental data (dark purple) are normalized before plotting, resulting in a Pearson correlation coefficient between simulation and experimental data being 0.9613 (A) and 0.9828 (B).

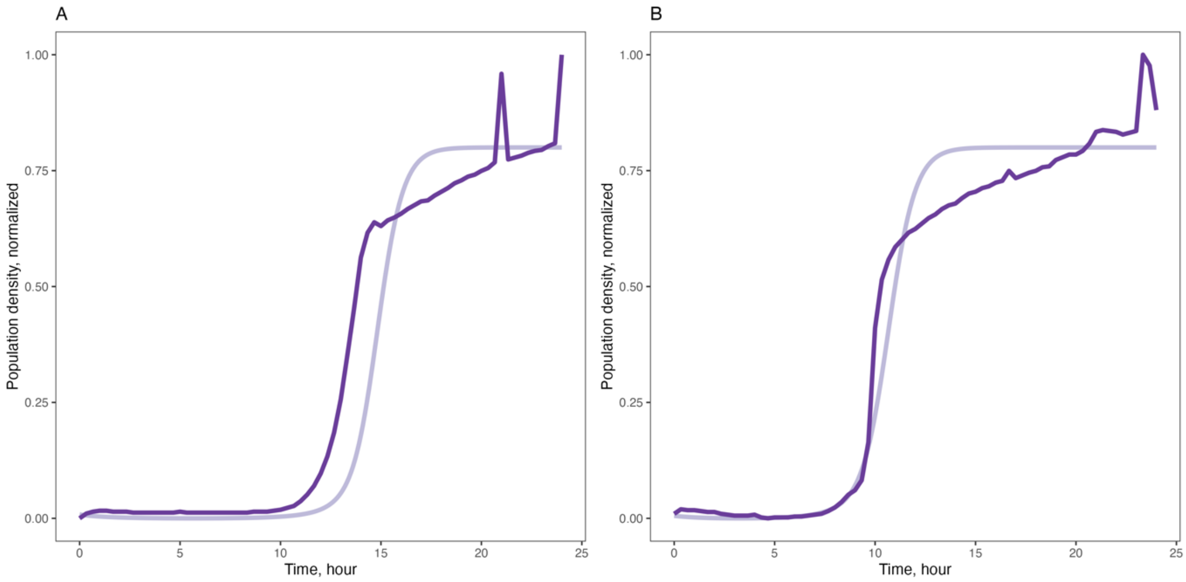

Fig S2. Simulation of a bacterial population growing in a serial passage. (A) Growth kinetics in OS-, OS+ environment and the decrease of H_2O_2 concentration in the OS+ environment. (B) Simulation of growth in a 1:100 serial passage.

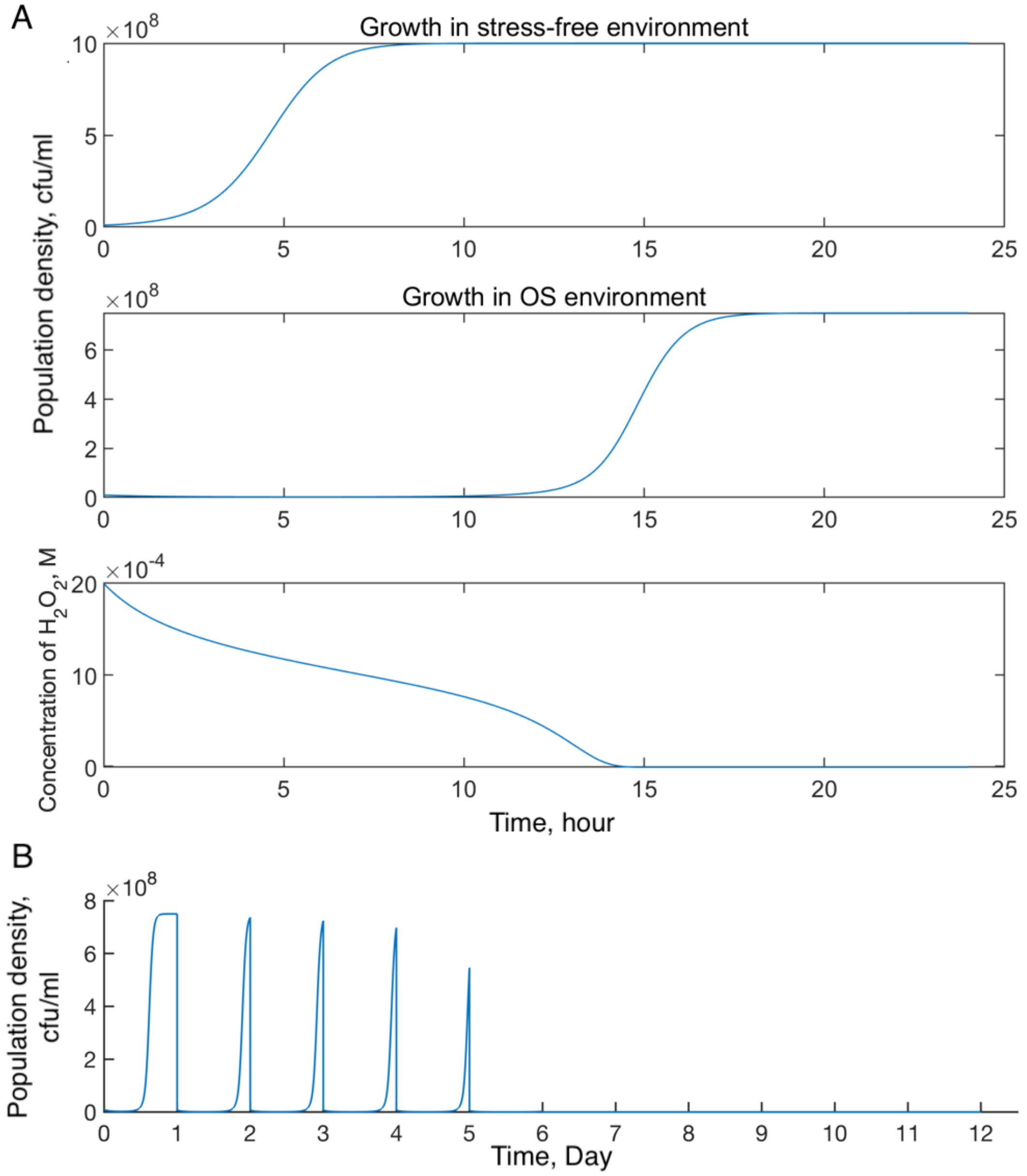

Fig S3. Simulation of bacterial growth of an evolved PA14 (fold change in sensitivity to H_2O_2 = 0.96) in 2 mM H_2O_2 environment (A) and a serial passage of 1:100 ratio with sensitivity to H_2O_2 reducing from wildtype PA14 to evolved PA14 on day 6 (B). (A) Both simulation data (light purple) and experimental data (dark purple) are normalized before plotting, resulting in a Pearson correlation coefficient between simulation and experimental data being 0.9836.

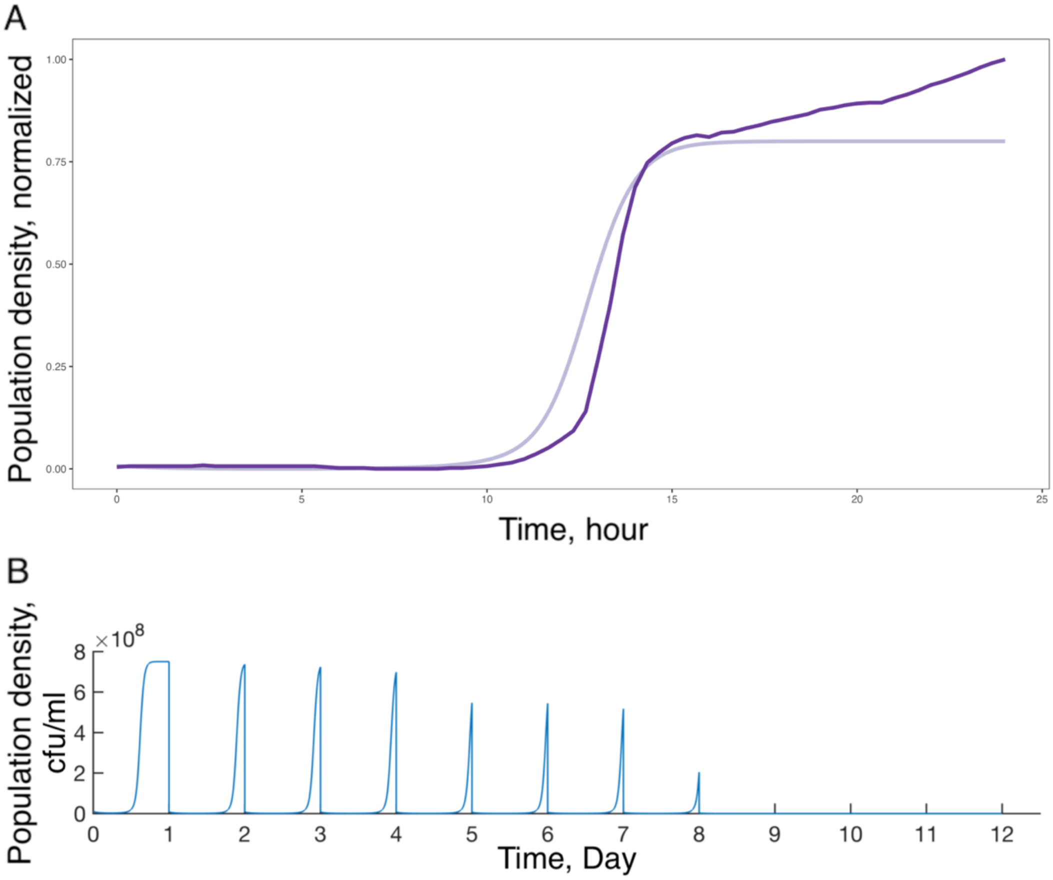

References:

1. Switala J, Loewen PC. Diversity of properties among catalases. Arch Biochem Biophys. 2002 May 15;401(2):145–54.

2. Srimahaeak T, Thongdee N, Chittrakanwong J, Atichartpongkul S, Jaroensuk J, Phatinuwat K, et al. *Pseudomonas aeruginosa* GidA modulates the expression of catalases at the posttranscriptional level and plays a role in virulence. Front Microbiol [Internet]. 2023 [cited 2023 May 14];13. Available from: https://www.frontiersin.org/articles/10.3389/fmicb.2022.1079710

3. Protein Data | NEB [Internet]. [cited 2023 May 14]. Available from: https://uk.neb.com/tools-and-resources/usage-guidelines/protein-data

4. Peroxy compounds: hydrogen peroxide and peroxyacetic acid environmental fate science chapter [Internet]. [cited 2023 Jun 9]. Available from: https://www3.epa.gov/pesticides/chem_search/cleared_reviews/csr_PC-000595_12-Jul-07_a.pdf
